# A Serosurvey Identifying Vulnerability to Measles in Health Care Workers. A Hospital-Based Prospective Seroprevalence Study

**DOI:** 10.3390/ijerph17124219

**Published:** 2020-06-12

**Authors:** Jana Malinová, Marek Petráš, Alexander M. Čelko

**Affiliations:** 1 Third Faculty of Medicine, Charles University in Prague, 10000 Prague, Czech Republic; jana.malinova@fnkv.cz (J.M.); martin.celko@lf3.cuni.cz (A.M.Č.); 2 Faculty Hospital Kralovské Vinohrady, 10000 Prague, Czech Republic

**Keywords:** serosurvey, seroprevalence, measles antibodies, persistence, health care workers

## Abstract

The aim of this serological survey was to assess the persistence of measles antibodies among health care workers (HCWs) at risk of incidental measles. A prospective study of measles-specific antibodies in serum samples obtained from a total of 2782 participants aged 19–89 years was conducted between May 2018 and December 2019. The seropositivity rate of 93.7% (95% CI: 92.4–94.9%) in fully vaccinated participants aged 19–48 years was significantly lower than that of 98.0% (95% CI: 96.5–99.0%) in participants naturally immunised before 54 years. A cohort of those born in 1971–1975, vaccinated predominantly with one dose, showed lower seropositivity persistence (86.6%) than those fully vaccinated with two doses or naturally immunised. Otherwise, seropositivity was not markedly influenced by sex, age, smoking status, overweight, obesity or concomitant disease. The presence of sufficient antibody levels in a high proportion of HCWs irrespective of the way they acquired immunity is a favourable finding for managing incidental measles; hence, in the presence of a risk of a measles outbreak, it would be possible to perform targeted vaccination of only at-risk HCWs with a history of incomplete vaccination or missing information about the way in which immunity is acquired.

## 1. Introduction

Measles is a highly contagious disease caused by morbillivirus. It is reported that up to 90% of susceptible persons could be infected upon contact with a patient diagnosed with measles. The measles virus can be transmitted directly through droplets and can survive up to two hours in an airspace where the patient sneezed or coughed. Moreover, an infected person can pose a risk for others four days before the onset of clinical manifestations as well as four days after symptoms disappear [1].

Measles remains one of the leading causes of child morbidity and mortality worldwide with the greatest burden borne by the youngest children [2,3]. Therefore, the implementation of universal vaccination programs, including measles vaccinations, is a most effective tool for its prevention. Despite stepped up efforts, measles has become a public health challenge of our century. Obviously, one of the factors can be the indifference or even reluctance of parents to have their offspring vaccinated. Yet another factor not to be underestimated is the persistence of antibodies against measles that can wane with time. Therefore, a continuous follow-up of sero-immunity is conducted worldwide.

In the Czech Republic, routine vaccinations against measles were introduced with a single-dose administration in 1969 to be expanded by a two-dose schedule in 1975. The original monovalent measles vaccine was replaced by a combination vaccine against measles, mumps and rubella in 1995. The vaccination rates in children <2 years old has ranged between 96 and 99% for the first dose, and between 84 and 99% for the second dose in the last decade [4]. The lower second-dose vaccination rate in the year 2018 was because of its intentional re-scheduling to the age of 5 years. Overall, the vaccination rate of the Czech pediatric population with at least one dose of vaccine remained high and presumably sufficient to contain the spread of the measles infection in the population. Despite this, a total of 943 cases of measles, especially in the adult population, were reported in the Czech Republic between 2017 and 2019.

As reported in many studies in the relevant literature, insufficient sero-protection in health care workers (HCWs) can pose a risk both for themselves and for patients [5,6,7]. Even though several observations documented >90% seroprevalence against measles among HCWs in Italy [8], France [6], Spain [9] or Japan [10], no similar study has been conducted in the Czech Republic. Therefore, this fact served as an impetus to design and conduct a serological survey among health care workers of the Prague-based Královské Vinohrady University Hospital between May 2018 and December 2019. The primary objective was to evaluate the measles antibody status among HCWs and to assess their possible vulnerability to the disease. Moreover, we attempted to find possible factors influencing HCWs seroprevalence.

## 2. Materials and Methods

Our prospective seroprevalence study was conducted among participants predominantly recruited from health care workers of the Faculty hospital Královské Vinohrady, in Prague. Upon receiving the consent of the would-be participants, their year of birth, weight and height, concomitant treatment and smoking status were obtained from the hospital staff database.

The participants were interviewed to determine whether or not they had been immunised or had had measles anytime in their lifetime. All declared either vaccination against measles or measles infection in childhood. As their original immunisation status could not be verified, the participants were further divided into 5-year age cohorts according to the year of birth (Table 1). The stratified cohorts were further assessed according to the history of routine vaccination against measles in the Czech Republic. The cohorts of participants born before 1966 were considered to be naturally immunised against measles while those born after 1976 were viewed as fully vaccinated with two doses of the measles vaccine. Participants born between 1966 and 1975 were either naturally immunised or vaccinated with one or two doses. 

Prior to their enrolment in the study, the participants were thoroughly informed about the study and signed informed consent forms. The study protocol was approved by the Ethics Committee of the Third Faculty of Medicine, Charles University in Prague (N° 0120/17).

The current sero-immunity status of participants was assessed by serological analysis of serum samples that had been collected, stored at below −20 °C and processed in one central laboratory (Laboratory of Clinical Immunology, Královské Vinohrady University Hospital, Prague, Czech Republic). The levels of antibodies were determined using a measles IgG ELISA kit (Immunolab GmbH, Kassel, Germany). The seropositivity cut-off of >12 AU/mL (arbitrary units per millilitre) was adopted from marginal positivity declared by the serological kit manufacturer [11].

Persistence of specific antibodies against measles was assessed both by the seropositivity rate, defined as the proportion of participants having antibody levels higher than the cut-off value, and by the geometric mean concentrations (GMCs) of measles antibodies. The demographic, behavioural and health characteristics were considered possible factors that could influence seropositivity persistence. Therefore, participants’ antibody levels or seropositivity were assessed in groups categorised by these factors. The differences in the GMCs between two groups or among several groups were investigated with the parametric t-Student test or by Dunnett multiple comparisons testing following one-way analysis of variance (ANOVA) after the log-transformation of antibody levels that met the normal distribution. Categorial variables were represented by the proportion including a 95% confidence interval computed by the method of Clopper and Pearson.

The differences in seropositivity rates were statistically evaluated with Fisher’s exact test, including crude odds ratios (cORs) obtained from the 2 × 2 table. The sample size was justified for logistic regression with 7 covariates—investigated predictors—that provided the adjusted odds ratios (aORs) including a 95% confidence interval (95% CI). Moreover, McFadden’s R squared as well as the *p*-value of this approach were sufficient for the predictive ability of this model.

All tests were two-tailed, and the level of significance was set at 0.05. Statistical analyses and logistic regression were performed using Prism 8 (GraphPad Software, Inc., San Diego, CA, USA) and STATA version 16 software (StatCorp, Lakeway Drive, TX, USA), respectively.

## 3. Results

Between May 2018 and December 2019, serum samples were obtained from a total of 2784 subjects with a mean age of 42 years (95% CI: 41.5–42.5 years). Although the participants were mostly women (74.7%), the age means for men and women did not differ relevantly (42.7 years for females and 40.1 years for males).

Body mass index (BMI) was not recorded in a total of 1039 subjects, i.e., 37.3% (95% CI: 35.5–39.1%), and was unavailable, especially in women (69%). The mean of BMI was significantly higher in men (26.5 kg/m^2^) than in women (25.2 kg/m^2^) but the difference did not reach clinical relevance. Almost 53% of subjects with known BMI had a normal body weight <25 kg/m^2^, i.e., 33.2% of all enrolled participants. The distribution of participants by the BMI category is shown in Table 1.

A concomitant disease was present in 41.1% of subjects having been diagnosed most often with endocrine, nutritional and metabolic diseases (13%)—especially thyroid gland diseases or impaired lipoprotein metabolism, and cardiovascular disease (8%)—especially hypertension heart disease; or respiratory diseases (6%).

The survey participants were recruited mostly from among hospital medical staff (80.6%; broadly defined as physicians, nurses and allied health professionals) and among hospital support (laundry, cleaning, administrative, etc.) staff, with the latter group including 2.4% of foreign nationals. Information about cigarette smoking vs. non-smoking status was missing in 50% of the serologically assessed subjects.

The youngest cohort, aged 19–23 years (years of birth 1996–2000), made up 6% of the whole group. Larger cohorts comprised those born in 1976–80 and 1971–75 (participants aged 39–43 years and 44–48 years, respectively). Fully vaccinated participants were defined as those born after the year of 1975 (1510 subjects). Conversely, 600 participants with naturally acquired immunity were assigned to age cohorts <1966.

Seropositivity against measles was detected in 93.5% of participants (95% CI: 92.6–94.4%) independently of sex, age, comorbidities, and other investigated factors (Table 2). The overall GMCs in all participants achieved 16.3 AU/mL (95% CI: 16.1–16.6 AU/mL), differing significantly between seronegative (8.5 AU/mL; 95% CI: 8.1–8.9 AU/mL) and seropositive participants (17.2 AU/mL; 95% CI: 17.0–17.4 AU/mL).

The seropositivty rate in the cohorts fully immunised with vaccine only (participants aged 19–43 years) was 93.7% (95% CI: 92.4–94.9%). Conversely, 98.0% (95% CI: 96.5–99.0%) of those naturally immunised by measles maintained their seropositivity longer than 54 years. Naturally acquired immunity against measles persisted in significantly more subjects than immunity induced by a vaccine, as demonstrated by an odds ratio of 3.29 (95% CI: 1.79–6.04). Likewise, the GMCs of measles antibodies were significantly higher in participants who had had measles (20.7 AU/mL; 95% CI: 20.1–21.3 AU/mL) than in those fully vaccinated (15.3 AU/mL; 95% CI: 15.1–15.5 AU/mL) or in those having received at least one vaccine dose (15.2 AU/mL; 95% CI: 15.0–15.4 AU/mL).

The seropositivity rate for measles did not differ between males and females although the GMCs of antibodies were significantly higher in women (Table 2). A sensitivity analysis demonstrated that the difference in the GMCs between males and females depended on of the way in which immunity is acquired. While the persistence of naturally acquired antibody levels did not differ between both sexes, vaccinated women had significantly higher GMCs of measles antibodies (16.1 AU/mL; 95% CI: 15.1–15.6 AU/mL) than vaccinated men (14.8 AU/mL; 95% CI: 14.4–15.2 AU/mL), with a *p*-value of 0.036.

The time since childhood vaccination did not influence the persistence of antibody levels as no difference in seropositivity rates between the two-dose vaccinated cohorts was found, i.e., the 5-year cohorts since the year of 1976 did not exhibit different seropositivity rates. Participants born in the 1971–1975 period, immunised predominantly with a single vaccine dose, achieved a seropositivity rate of 86.6% (95% CI: 82.8–89.9%), a value significantly lower compared with that seen in the youngest, fully vaccinated participants (i.e., 94%; 95% CI: 89.3–97.1%).

The survey did not find an impact of BMI on the persistence of seropositivity rates, which did not differ among the categories of normal weight, overweight, obesity or severe obesity. The antibody levels remained consistent across all BMI categories, as demonstrated by their similar GMCs. Moreover, sensitivity analysis confirmed consistent seropositivity rates stratified by BMI categories both in fully vaccinated participants and those naturally immunised by measles.

The persistence of seropositivity rates was similar in smokers and non-smokers irrespective of the way in which immunity had been acquired. Unknown smoking status in 1381 participants was associated with lower seropositivity rates as well as GMCs compared to those of non-smokers (Table 2). This difference was confirmed only in naturally immunised participants (aOR = 0.36; 95% CI: 0.20–0.67).

No difference in serological persistence was observed in participants with or without concomitant disease, as demonstrated by their seropositivity rates and the GMCs of measles antibodies.

Likewise, the seropositivity rates in patients with endocrine, nutritional or metabolic diseases (93.7%; 95% CI: 90.6–96.0%) and in those with cardiovascular disease (92.7%; 95% CI: 88.5–95.8%) did not differ from those of healthy participants. The sensitivity analyses showed lower seropositivity rates in naturally immunised participants with any concomitant disease (97.3%; 95% CI: 94.8–98.8%) than in those without it (98.7%; 95% CI: 96.6–99.6%) as documented by an aOR of 0.17 (95% CI: 0.03–0.88). There was no difference in the persistence of seropositivity rates or GMCs of measles antibodies between hospital medical staff and hospital support staff as defined above, as well as between foreign nationals and Czech citizens.

## 4. Discussion

The seropositive levels of measles antibodies were observed in almost all (94%) of the survey participants, especially in HCWs, irrespective of the way in which immunity was acquired. This value is close to that reported for adult populations reported by seroprevalence studies in other countries [12,13], in the Czech Republic in 2013 [14] as well as studies conducted in HCWs [6,8,9,10]. Even though these findings are favourable, it does not mean that they will remain permanent since serological surveys conducted in Italy (in 2003–2019) showed a steep decline in seropositivity rates induced by measles vaccinations that ranged between 77.2 and 84.4% in the population younger than 50 years [15,16,17].

We found that the persistence of seropositivity rates was not dependent on the time since acquisition of immunity, but was determined by the way in which immunity was acquired, i.e., if the seropositivity was induced by vaccination or by childhood measles. Almost all of the participants (98%) with naturally induced antibodies against measles in childhood maintained higher antibody levels (20.7 AU/mL) than those fully or partially vaccinated (15.2 AU/mL). As no significant variation in seropositivity rates was observed among the 5-year cohorts of naturally immunised participants (range, 97–100%), naturally acquired immunity could presumably persist at least 54–70 years. This was in accordance with the rates used in modelling measles re-emergence [18].

In line with published data, long-term seropositivity induced by full vaccination was present in almost 94% participants aged 19–43 years [12,13,14]. This value differed significantly from that of participants born in the 1971–1975 period, the majority of whom received only a single dose of the measles vaccine (86.6%). It was consistent with a previous finding that the Czech population born between 1968 and 1978 exhibited a lower seroprevalence of 80.4% compared to a younger or older study population [14].

Our survey did not identify a single clinically relevant factor influencing the persistence of seropositivity. Even if slightly higher GMCs of antibodies were observed in women than in men, the difference, about 1 AU/mL, was non-significant. While concomitant diseases did not change the seropositivity rates in vaccinated participants, they may have contributed to a small decrease in seropositivity persistence in naturally immunised individuals. Again, it was not clear whether this finding was of clinical importance because the rate difference was less than 2%. Otherwise, the seropositivity rates in smokers, overweight and obese individuals as well as the hospital support staff did not differ significantly from those of the study population based on the way in which immunity was acquired.

It follows from the above that the persistence of antibodies against measles is determined primarily by the way in which immunity is acquired. The vaccine-induced humoral immunity, persisting 20 years or longer, was always shorter than the persistence of naturally acquired immunity, with the reduction likely occurring within the first 10–15 years after full vaccination [19,20]. Nevertheless, the seropositive levels of antibodies were observed in more than 87% of those vaccinated with at least one dose within 48 years.

The question yet to be answered is whether or not the observed sero-persistence is sufficient to counter measles resurgence. Unfortunately, the results of our study are not able to provide the answer since further immune mechanisms and factors such as complement, immunological memory, and cellular immunity play an important role in one’s protection [21]. Furthermore, the threshold of enzyme immunoassay for determining protective antibody levels has not been yet standardised [22,23].

Furthermore, our results may have been biased by indirect booster immunisation due to an exposure to measles, especially in those born in the 1970–1985 period, which we hope is not the case. Even if the main study population was that of hospital medical staff, less than 20% of participants were enrolled from among hospital support staff. A selection bias with this small subgroup could not have a major impact on our results, as demonstrated by the similar seropositive rates. A potential limitation of the present serological survey was the lack of immunisation status verification—our inability to verify the immunization status of our study participants, which was only self-reported.

However, we are not convinced that fully vaccinated or naturally immunised health care workers need to be systematically revaccinated by one or more vaccine doses in adult age. Moreover, the outcome of our study in HCWs was similar to that of another survey conducted in the Czech general population in 2013, making it unlikely that this specific subgroup of individuals would be at higher risk than the rest of the population.

## 5. Conclusions

A full measles vaccination in childhood was capable of inducing sufficient antibody levels that persisted at seropositive values that most of the health care workers acquired over a period of 43 years. As expected, the levels of vaccine-induced antibodies were lower than those acquired naturally. Despite this, we recommend that, instead of systematic revaccination or the serological monitoring of antibodies, targeted immunisation of at-risk health care workers with incomplete vaccinations or with missing information about their sero-immunity status could prove a quick, highly effective and reasonable tool in the event of a measles outbreak.

## Figures and Tables

**Table 1:** Number and proportion of participants (including the 95% confidence intervals) stratified by assessed demographic, behavioural and health factors.

Stratification		All		Women	
		N ^1^	Proportion (%); 95% CI ^3^	N	Proportion (%); 95% CI
Participants		2784		2080	74.7 (73.1–76.3)
Age cohort	1996–2000	168	6.0 (5.2–7.0)	115	68.5 (60.8–75.4)
	1991–1995	354	12.7 (11.5–14.0)	229	64.7 (59.5–69.7)
	1986–1990	298	10.7 (9.6–11.9)	206	69.1 (63.5–74.3)
	1981–1985	299	10.7 (9.6–11.9)	217	72.6 (67.1–77.6)
	1976–1980	391	14.0 (12.8–15.4)	308	78.8 (74.4–82.7)
	1971–1975	389	14.0 (12.7–15.3)	300	77.1 (72.6–81.2)
	1966–1970	285	10.2 (9.1–11.4)	236	82.8 (77.9–87.0)
	1961–1965	242	8.7 (7.7–9.8)	202	83.5 (78.2–87.9)
	1956–1960	207	7.4 (6.5–8.5)	167	80.7 (74.6–85.8)
	1951–1955	83	3.0 (2.4–3.7)	66	79.5 (69.2–87.6)
	<1950	68	2.4 (1.9–3.1)	34	50.0 (37.6–62.4)
BMI ^2^ (kg/m^2^)	<25	924	33.2 (31.4–35.0)	760	82.3 (79.6–84.7)
	25–<30	512	18.4 (17.0–19.9)	360	70.3 (66.1–74.2)
	30–<35	246	8.8 (7.8–10.0)	195	79.3 (73.7–84.2)
	≥35	63	2.3 (1.7–2.9)	45	71.4 (58.7–82.1)
	Unknown	1039	37.3 (35.5–39.1)	720	69.3 (66.4–72.1)
Concomitant disease		1144	41.1 (39.3–42.9)	940	82.2 (79.8–84.3)
Health care worker		2243	80.6 (79.0–82.0)	1699	75.7 (73.9–77.5)
Foreign national		68	2.4 (1.9–3.1)	36	52.9 (40.4–65.2)
Smoker	No	882	31.7 (30.0–33.4)	709	80.4 (77.6–83.0)
	Yes	521	18.7 (17.3–20.2)	410	78.7 (74.9–82.1)
	Unknown	1381	49.6 (47.7–51.5)	961	69.6 (67.1–72.0)

^1^ N: number of participants; ^2^ BMI: body mass index; ^3^ CI: confidence intervals.

**Table 2:** Persistence of seropositivity against measles and geometric mean concentrations (GMCs) of measles antibodies, crude and mutually adjusted odds ratios (cORs, aORs) including the 95% confidence interval.

Predictors		N ^1^	n ^2^	Seropositivity %	cOR ^3^	aOR ^4^ (95% CI)	GMC ^5^ (95%CI); AU/mL ^6^
Total		2784	2604	93.5 (92.6–94.4)			16.3 (16.1–16.6)
Sex	Male	704	655	93.0 (90.9–94.8)	1	1	15.7 (15.3–16.1)
	Female	2080	1949	93.7 (92.6–94.7)	1.11 (0.79–1.56)	1.13 (0.80–1.62)	16.6 (16.3–16.8) ^†^
Age cohort	1996–2000	168	158	94.0 (89.3–97.1)	1	1	14.5 (13.9–15.2)
	1991–1995	354	337	95.2 (92.4–97.2)	1.25 (0.56–2.80)	1.17 (0.52–2.65)	15.9 (15.4–16.5)
	1986–1990	298	285	95.6 (92.7–97.7)	1.39 (0.59–3.24)	1.29 (0.53–3.13)	16.0 (15.4–16.6)
	1981–1985	299	271	90.6 (86.8–93.7)	0.61 (0.29–1.29)	0.55 (0.25–1.21)	14.5 (14.0–15.0)
	1976–1980	391	364	93.1 (90.1–95.4)	0.85 (0.40–1.80)	0.78 (0.35–1.72)	15.0 (14.6–15.5)
	1971–1975	389	337	86.6 (82.8–89.9)	0.41 (0.20–0.83) *	0.37 (0.18–0.78) *	14.7 (14.2–15.2)
	1966–1970	285	264	92.6 (89.0–95.4)	0.80 (0.37–1.73)	0.75 (0.33–1.71)	16.6 (16.0–17.4) ^†^
	1961–1965	242	235	97.1 (94.1–98.8)	2.12 (0.79–5.70)	1.94 (0.70–5.45)	20.9 (19.8–22.0) ^‡^
	1956–1960	207	202	97.6 (94.5–99.2)	2.56 (0.86–7.63)	2.43 (0.79–7.49)	20.8 (19.8–21.9) ^‡^
	1951–1955	83	83	100.0 (95.7–100.0)	x	x	20.8 (19.5–22.3) ^‡^
	<1950	68	68	100.0 (94.7–100.0)	x	x	19.2 (18.0–20.5) ^‡^
BMI ^7^ (kg/m2)	<25	924	863	93.4 (91.6–94.9)	1	1	16.3 (16.0–16.7)
	25–<30	512	478	93.4 (90.8–95.4)	0.99 (0.64–1.53)	0.98 (0.63–1.53)	16.6 (16.1–17.2)
	30–<35	246	228	92.7 (88.7–95.6)	0.90 (0.52–1.55)	0.88 (0.50–1.53)	16.6 (15.9–17.4)
	≥35	63	60	95.2 (86.7–99.0)	1.41 (0.43–4.64)	1.41 (0.42–4.75)	16.1 (15.0–17.3)
	Unknown	1039	975	93.8 (92.2–95.2)	1.08 (0.75–1.55)	1.56 (0.99–2.47)	16.2 (15.8–16.5)
Concomitant disease	No	1640	1539	93.8 (92.6–95.0)	1	1	16.2 (16.0–16.5)
	Yes	1144	1065	93.1 (91.5–94.5)	0.88 (0.65–1.20)	0.83 (0.59–1.16)	16.5 (16.1–16.9)
Health care worker	No	541	501	92.6 (90.1–94.7)	1	1	15.9 (15.5–16.4)
	Yes	2243	2,103	93.8 (92.7–94.7)	1.20 (0.83–1.73)	1.27 (0.84–1.92)	16.4 (16.2–16.7)
Foreign national	No	2716	2540	93.5 (92.5–94.4)	1	1	16.3 (16.1–16.6)
	Yes	68	64	94.1 (85.6–98.4)	1.11 (0.40–3.08)	1.12 (0.37–3.43)	16.7 (15.3–18.3)
Smoker	No	882	838	95.0 (93.4–96.4)	1	1	16.8 (16.4–17.2)
	Yes	521	490	94.0 (91.7–95.9)	0.83 (0.52–1.33)	0.83 (0.51–1.33)	17.0 (16.5–17.5)
	Unknown	1381	1276	92.4 (90.9–93.7)	0.64 (0.44–0.92) ^‡^	0.48 (0.31–0.72) ^‡^	15.9 (15.6–16.2) ^†^

^1^ N: total number of factor–stratified participants; ^2^ n: total number of seropositive participants; ^3^ cOR: crude odds ratio; ^4^ aOR: mutually adjusted odds ratio; ^5^ GMC: geometric mean concentration; ^6^ AU/mL: arbitrary units per millilitre; ^7^ BMI: body mass index; * *p* < 0.05; ^†^ *p* < 0.001; ^‡^ *p* < 0.0001.
